# Lead-free epitaxial ferroelectric material integration on semiconducting (100) Nb-doped SrTiO_3_ for low-power non-volatile memory and efficient ultraviolet ray detection

**DOI:** 10.1038/srep12415

**Published:** 2015-07-23

**Authors:** Souvik Kundu, Michael Clavel, Pranab Biswas, Bo Chen, Hyun-Cheol Song, Prashant Kumar, Nripendra N. Halder, Mantu K. Hudait, Pallab Banerji, Mohan Sanghadasa, Shashank Priya

**Affiliations:** 1 Center for Energy Harvesting Materials and Systems (CEHMS), Department of Mechanical Engineering, Virginia Tech, Blacksburg, Virginia 24061, USA; 2 Advanced Devices & Sustainable Energy Laboratory (ADSEL), Bradley Department of Electrical and Computer Engineering, Virginia Tech, Blacksburg, Virginia 24061, USA; 3 Materials Science Centre, Indian Institute of Technology Kharagpur, Kharagpur 721302, India; 4 Advanced Technology Development Centre, Indian Institute of Technology Kharagpur, Kharagpur 721302, India; 5 U.S. Army Aviation & Missile Research Development & Engineering Center (AMRDEC) Redstone Arsenal, Huntsville, AL 35898, USA

## Abstract

We report lead-free ferroelectric based resistive switching non-volatile memory (NVM) devices with epitaxial (1-x)BaTiO_3_-xBiFeO_3_ (x = 0.725) (BT-BFO) film integrated on semiconducting (100) Nb (0.7%) doped SrTiO_3_ (Nb:STO) substrates. The piezoelectric force microscopy (PFM) measurement at room temperature demonstrated ferroelectricity in the BT-BFO thin film. PFM results also reveal the repeatable polarization inversion by poling, manifesting its potential for read-write operation in NVM devices. The electroforming-free and ferroelectric polarization coupled electrical behaviour demonstrated excellent resistive switching with high retention time, cyclic endurance, and low set/reset voltages. X-ray photoelectron spectroscopy was utilized to determine the band alignment at the BT-BFO and Nb:STO heterojunction, and it exhibited staggered band alignment. This heterojunction is found to behave as an efficient ultraviolet photo-detector with low rise and fall time. The architecture also demonstrates half-wave rectification under low and high input signal frequencies, where the output distortion is minimal. The results provide avenue for an electrical switch that can regulate the pixels in low or high frequency images. Combined this work paves the pathway towards designing future generation low-power ferroelectric based microelectronic devices by merging both electrical and photovoltaic properties of BT-BFO materials.

**T**he resistive random access memory (ReRAM) has received significant research attention for non-volatile (NVM) memory device applications beyond 10 nm technology node. The component is also known as memristor, a fourth fundamental passive circuit element, which could open new design topologies^1^. The speed of ReRAM is about 10 ns, which is 100× faster than that of flash memory devices^2^. The development of ReRAM relies upon proper selection of materials including oxides, organics, solid electrolytes, and ferroelectrics^3^,^4^,^5^,^6^,^7^,^8^,^9^,^10^,^11^. However, the resistive effects in solid electrolytes based ReRAMs are usually based on electroforming or chemical process, which is a destructive process as it involves chemical reaction that could lead to thermal damage of the devices^11^. On the other hand, ferroelectric materials have attracted enormous attention due to their remnant polarization and polarization inversion^8^,^11^,^12^. No electroforming or chemical process takes place during the operation of ferroelectric ReRAM, thus enabling longer lifetime and faster operation. The data is stored, written or read by switching or inverting the polarization^8^,^11^,^12^. Polarization represents the net dipole moment in the ferroelectric and the switching phenomenon is generally discussed in terms of domains which represent the local distribution of dipoles with alignment along the applied field direction^12^,^13^. A good ferroelectric material can be easily poled by applying electric field above the coercive field^12^,^14^. This attribute of aligning the direction of dipoles through external field provides an opportunity to develop low-power devices.

Multiferroic BiFeO_3_ (BFO) film has been grown on different substrates to investigate its switching behaviour for ferroelectric ReRAM applications^7^,^8^,^9^,^10^,^11^. BFO is attractive due to its simultaneous ferroelectric and antiferromagnetic characteristics exhibiting both high Curie temperature and Neel temperature^15^. Several researchers have conducted investigation on the BFO based materials grown on SrRuO_3_^8^, Nb-doped SrTiO_3_ (Nb:STO)^9^, as well as Pt-coated Si substrate^11^. The studies on retention and endurance behaviour of fabricated BFO based ReRAM devices are very limited due to its high leakage characterized by increase in dielectric loss with applied electric field. Another essential device parameter is the high ON/OFF ratio which indicates a fast reliable operation^16^,^17^. The very high coercive voltage observed in BFO based ReRAM devices limits its applicability for low-power memory applications^8^,^9^,^11^,^18^. To address these limitations, several researchers have attempted to modify the structural properties of BFO by doping on the Bi site^19^,^20^,^21^ through synthesis of single phase solid solutions^22^. Since the leakage is mainly related to the presence of multivalent Fe ions in BFO so doping is convenient method for modulating the defect chemistry and thereby increasing the concentration of Fe^3+^ ions^15^,^18^,^22^. Prior studies on lead-free (1-x)BaTiO_3_ – xBiFeO_3_ (BT-BFO) solid solution have *mainly reported* the piezoelectric, dielectric, magnetic and magnetoelectric properties at room temperature so the magnitude of NVM relevant parameters is not known^15^,^23^. While Luo *et al.*^18^ have investigated the resistive switching in Mn-doped BFO, their study did not extend into the write-read operations. Thus, limited information is available in literature on suitability of BFO based compositions for the NVM application.

Another intriguing aspect of BFO based materials is related to its photovoltaic properties^9^,^24^,^25^,^26^,^27^. Although many studies have been conducted on developing ferroelectric based solar cells but the efficiencies are still much below the practical requirements. However, little attention has been paid to address the ultraviolet (UV)-detection properties of BFO or other ferroelectrics^28^,^29^,^30^,^31^. Most of the prior investigations have focused upon the spectral response of the ferroelectric materials when irradiated with different wavelengths of UV-light rather than understanding the time dependent detection^28^,^29^,^30^,^31^. In general, the UV-detection properties are limited to large bandgap semiconductors, such as GaN, AlN, and ZnO^32^,^33^. However, these materials (i) are expensive and complex to fabricate and (ii) suffer from deteriorating electrical performance over time^34^. Here, we address both these aspects by providing understanding of the ultraviolet response of the BFO based system and present promising results.

This paper reports the results on lead-free epitaxial (1-x)BT-xBFO (x = 0.725) thin film deposited on Nb:STO by pulsed laser deposition (PLD) that exhibits (i) higher retention time, (ii) higher endurance, (iii) low leakage current, (iv) fast switching response, (v) lower coercive voltage, (vi) higher ON/OFF current ratio, and (vii) reliable operation. We were able to achieve these attributes by carefully controlling the composition, growing the high quality film, and lowering the defect density. Piezoelectric force microscopy (PFM) measurement was utilized to study the ferroelectricity at room-temperature and also to demonstrate the write-read operation of memory devices. X-ray photoelectron spectroscopy (XPS) was used to determine the band alignment of BT-BFO/Nb:STO heterojunction and the results are used to explain the operating principle of NVM devices based on BT-BFO. Staggered gap band alignment between the BT-BFO and Nb:STO were achieved, which is favourable for photovoltaic and detector applications due to their ability for long-range photo induced charge separation^35^. We show the efficient UV-photovoltaic detection properties of BT-BFO/Nb:STO devices along with low response time. Our results open the possibility to develop optical read-write memories based upon the photovoltaic properties in BT-BFO^36^. We also demonstrate that our fabricated device can be used as a high frequency rectifier, which retain polarization-modulated rectification^37^. Both low and high frequency signals are applied at the inputs of the device, and the outputs were recorded and compared with the commercial Si diode. The obtained results highlight the promise of BT-BFO/Nb:STO heterojunctions for multifunctional devices such as high-speed ReRAMs, optoelectronic sensors, and high/low-frequency signal processing.

## Results

### Investigations of crystal structure and ferroelectricity in BT-BFO thin films

**Figure 1:**
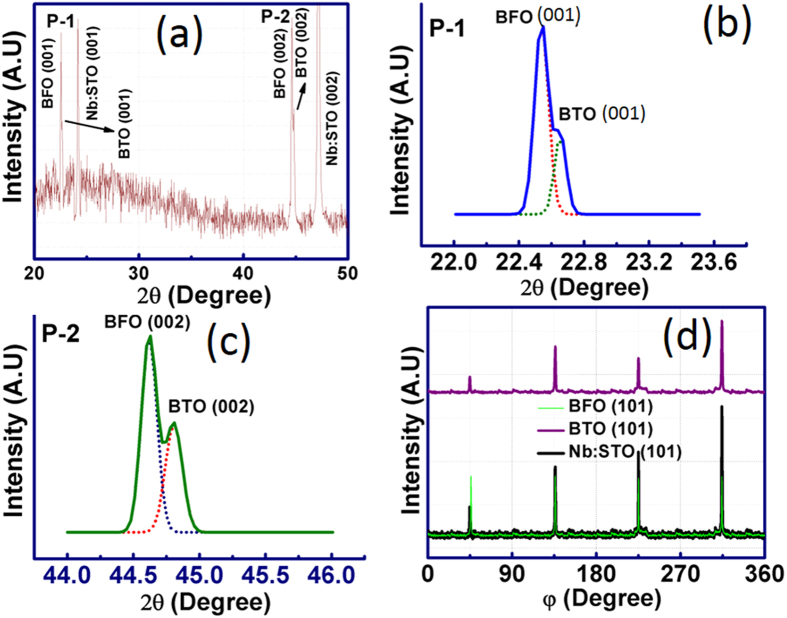
X-ray diffraction spectra of BT-BFO grown on Nb:STO. (**a**) High resolution θ – 2θ x-ray diffraction pattern of BT-BFO thin films grown on single crystalline (100) Nb:STO substrate. Zoomed-in images of (**b**) (100) BTO-BFO which is referred as Peak-1 (P1) and (**c**) (200) BTO-BFO which is referred as Peak-2 (P-2). There are no impurity peaks present in the XRD spectra and the film was found to be single crystalline. (**d**) φ- scan of BT-BFO thin films on Nb:STO. Four distinct peaks were observed separated by 90° indicating the fourfold symmetry, which demonstrates the formation of high quality epitaxial film.

The crystal structures of PLD grown BT-BFO films on Nb:STO substrates were characterized by x-ray diffraction (XRD) technique as shown in Fig. 1. From the Fig. 1(a) (θ – 2θ scan), it can be seen that only (001) and (002) peaks of the BTO, BFO and Nb:STO are present, indicating that both the BTO and BFO films are c-axis oriented with respect to normal to the substrate^38^. Figure 1(b,c) are the zoomed-in images of (100) BTO-BFO and (200) BTO-BFO, respectively. Phi (φ)-scans of (101) peaks were performed to ensure the epitaxy on the Nb:STO substrate (as shown in Fig. 1(d)). The epitaxial relationship can be represented as (001)BTO║(001)BFO║(001)Nb:STO and [101]BTO║[101]BFO║[101]Nb:STO. Four distinct peaks were observed separated by 90° indicating the fourfold symmetry which demonstrates the device quality epitaxial film^10^. It is interesting to note that there are no impurity peaks present in the XRD spectra which further confirms the formation of high quality films. Based upon our prior studies^38^, it has been found that both the ferroelectric and electrical properties can be improved when the BFO composition is greater than 0.50 in BT-BFO system.

**Figure 2:**
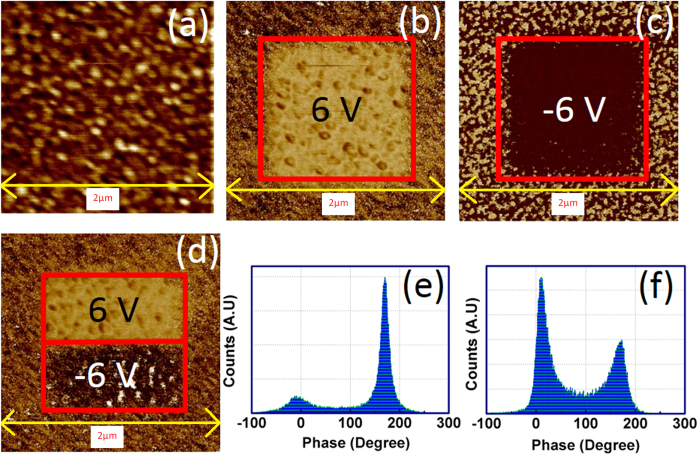
Ferroelectric domain switching observation. PFM micrographs of 50 nm BT-BFO thin films grown on Nb:STO. (**a**) surface topography, PFM phase micrograph after poling with (**b**) +6 V, (**c**) −6 V, and (**d**) ±6 V. From the colour contrast, it can be seen that the polarization inversions were achieved with opposite poling. Phase histogram of BT-BFO thin films after poling with (**e**) −6 V and (**f**) ±6 V.

**Figure 3:**
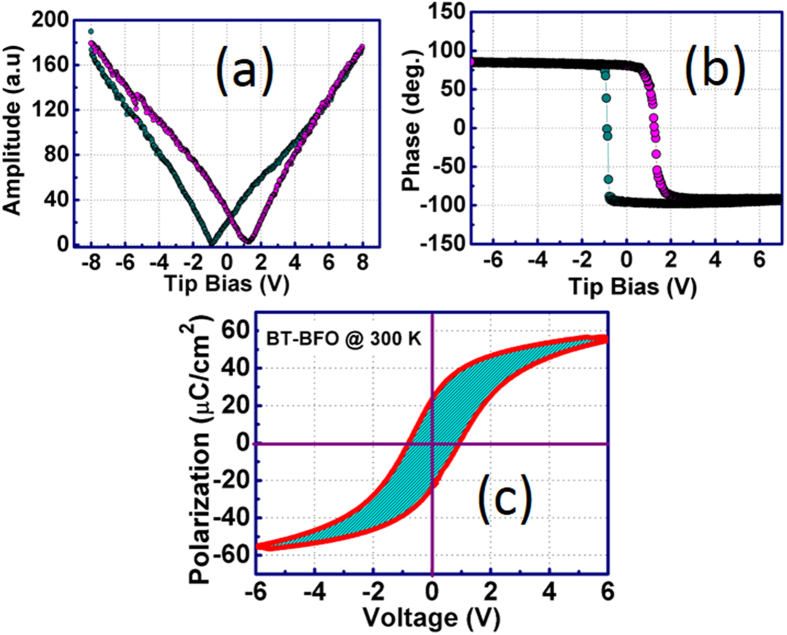
Local hysteresis behavior of BT-BFO thin films (thickness: 50 nm) on Nb:STO. Typical local hysteresis loops of BT-BFO measured by PFM (**a**) amplitude component; (**b**) polarization-voltage hysteresis loops; and (**c**) room-temperature polarization-voltage hysteresis loops using top electrode from electrical measurements. The material offers low coercive voltage and high remnant polarization, which are indispensable for low-power and high density non-volatile memory applications.

Figure 2(a) shows the PFM topography on 2 × 2 μm^2^ region of BT-BFO thin film deposited on Nb:STO. To understand the polarization switching, writing experiments were performed on 1 × 1 μm^2^ regions which were negatively (as shown in Fig. 2(b)) and positively poled (as shown in Fig. 2(c)), and read with 2 × 2 μm^2^ region after poling with specific bias. The change in the color contrast in the 1 × 1 μm^2^ region indicates the effective polarization switching. In order to demonstrate the direct polarization inversion, the 1 × 0.5 μm^2^ region was poled with +6 V and the other region with the same area was poled with −6 V followed by reading of 2 × 2 μm^2^ region. The typical micrograph is shown in Fig. 2(d). Two different color contrasts present in both the 1 × 0.5 μm^2^ regions provide the direct evidence for polarization inversion in BT-BFO. It is important to note that the demonstration of polarization inversion in ferroelectric material is extremely important in order to understand whether it has the capability for rewriting and changing states in NVM devices^13^. In our devices, the ferroelectric domains are not pinned and a complete polarization inversion is easily achieved in a repetitive manner. Thus, the results in Fig. 2 confirm that the BT-BFO exhibits desired read / write features. The phase histogram was recorded after dc writing on the BT-BFO film and is shown in Fig. 2(e,f). From Fig. 2(e), it can be seen that there was 180° phase shift after writing with positive bias which was also the case in Fig. 2(b). The 180° polarization inversion was observed when the film was poled with both negative and positive bias as shown in Fig. 2(d). The amplitude of the phase shift due to negative poled regions was found to be less when compared with the positive pole one. This may be due to the change in conductivity in these downward and upward polarization regions^10^. The PFM hysteresis loop both in amplitude and phase are recorded as a function of applied voltage and are shown in Fig. 3(a,b), respectively. Both the amplitude and phase plots consisted of a clear hysteresis, and a sharp 180° inversion at the coercive voltages can be observed. From Fig. 3(b), the coercive voltage was found to be −1 and 1.10 V. The polarization-voltage (P-V) hysteresis loop for BT-BFO thin films is shown in Fig. 3(c) measured at room temperature. Figure 3(c) depicts the ferroelectric square hysteresis loop for BT-BFO epitaxial films. The saturated polarization was found to be 23.5 μC/cm^2^, whereas, the coercive voltage was 1.10 V. From this result, we can expect lower leakage in BT-BFO films which is an important criterion for achieving high retention in NVM devices. It is noteworthy to mention that coercive voltage was consistent in both the electrical and PFM polarization-voltage measurements. We believe that the reason could be due to the use of same top electrodes in the NVM devices (Pt for both the electrical P-E and tip for PFM P-V)^39^,^40^.

### Study of the fabricated devices

**Figure 4:**
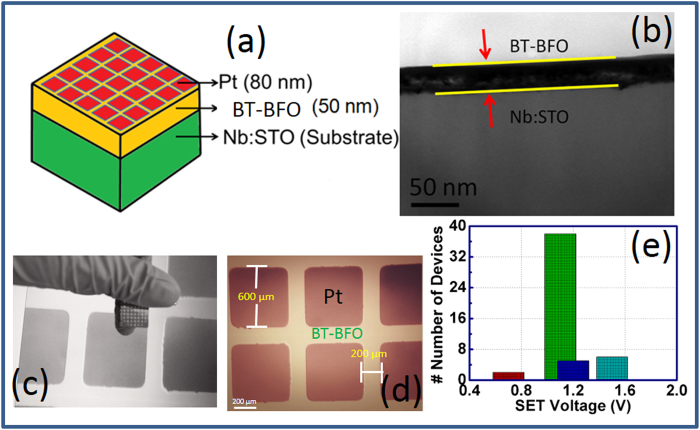
Fabricated Pt/BT-BFO/Nb:STO devices. (**a**) Schematic representation of BT-BFO resistive memory devices sandwiched between Pt top electrodes and Nb:STO. (**b**) Cross sectional transmission electron microscope image of BT-BFO and Nb:STO. The thickness of BT-BFO was found to be 50 nm. Optical microscope images of the (**c**) fabricated and (**d**) top view of Pt/BT-BFO/Nb:STO resistive memory devices. Pt top electrodes of dimension 600 μm × 600 μm are patterned onto Nb:STO substrates. The interval between contiguous electrodes are 200 μm. (**e**) The number of fabricated memory devices with different set voltages.

The schematic representation of BT-BFO based NVM memory devices is shown in Fig. 4(a). In this device structure, Pt was used as the top electrode and the BT-BFO film was sandwiched between the Pt and Nb:STO substrate. Indium (In) was used as a bottom electrode to make an ohmic contact for the electrical transport measurement. Cross-sectional transmission electron microscopy (TEM) micrograph of a representative 50 nm thin BT-BFO layer on Nb:STO substrate is shown in Fig. 4(b), confirming the uniform PLD deposited BT-BFO film with well-defined sharp interface. Figure 4(c) shows the optical micrograph of the fabricated NVM devices. We have fabricated 51 devices onto Nb:STO substrate where the separation between each device is 200 μm whereas the dimension of each Pt electrode is 600 × 600 μm^2^ (as shown in Fig. 4(d)). Out of 51 devices, 80% of the memory devices were found to perform as desired. The yield of Pt/BT-BFO/Nb:STO memory devices with different set voltages is shown in a histogram (Fig. 4(e)). Interestingly, the set voltage was found to be 1.10 V for majority of the devices, which is in agreement with the measured coercive voltage of ~1.1 V as shown in Fig. 4(a,c).

### Electrical characterization

**Figure 5:**
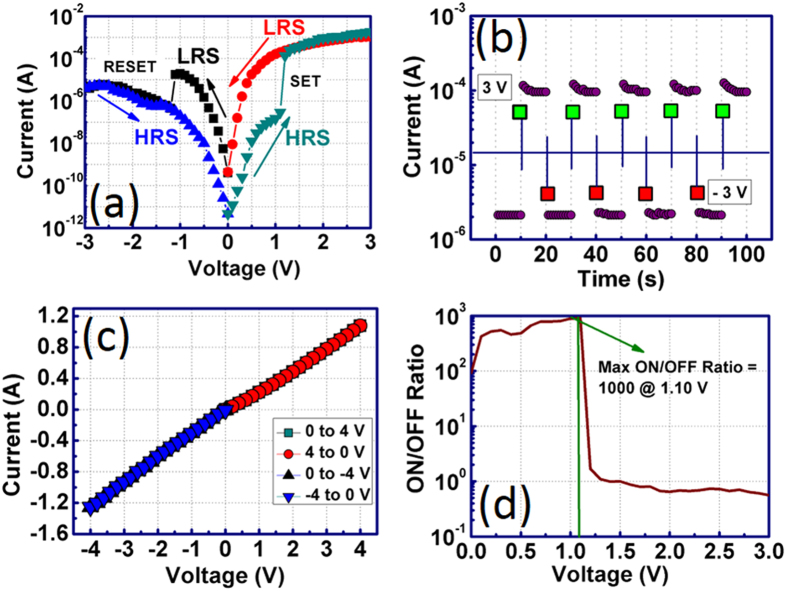
Current-voltage switching characteristics. (**a**) The I-V performances are measured by sweeping the voltage from 0 to +3 V, +3 to 0 V, 0 to −3 V, and −3 to 0 V. It was found that the current was in virgin to HRS up to 1.10 V and a sudden transition to LRS was noticed at 1.10 V, which was further considered as the coercive voltage. The device maintained in the LRS during the voltage was swept back to 0 V from 3 V. (**b**) Two states switching characteristics of the fabricated NVM devices. Two different voltages were applied across the devices and the current *versus* time was read. The device was completely switched and two different states are clearly distinguished. (**c**) The I-V performances of Pt/Nb:STO devices and the characteristics are found to be linear. Therefore, the memory property is originated when the BT-BFO was inserted between Pt and Nb:STO. (**d**) ON/OFF ratio of Pt/BT-BFO/Nb:STO devices. The ON/OFF ratio can be referred as the ratio of the current at LRS to the current at HRS. The variation of ON/OFF ratio at different voltages were recorded. The device offers high peak for ON/OFF ratio at 1.10 V.

The room temperature resistive switching characteristics of Pt/BT-BFO/Nb:STO memory devices is shown in Fig. 5(a). The voltage was varied from 0 to 3 V, 3 to 0 V, 0 to −3 V and −3 to 0 V, which were named as high resistance state (HRS), low resistance state (LRS), LRS, and HRS respectively. Hysteresis in I-V characteristics was observed in the BT-BFO/Nb:STO heterojunction devices. Initially, before applying any bias, the memory device was in the virgin state, i.e., in HRS. When the positive voltage was applied on the device, it was still in HRS until 1 V. It was found that the current suddenly increased from 5 × 10^−7^ A to 2 × 10^−4^ A at 1.10 V. Increase in this current at 1.10 V can be referred to as LRS and the 1.10 V is referred as a set voltage. The device was in LRS at higher positive voltages (1.10 to 3 V). At 3 V, the current was found to be 2 × 10^−3^ A. When the voltage was driven back from 3 to 0 V, the device still retained the LRS and the current at 1.10 V was found to be 2.20 × 10^−4^ A. The device can be switched from LRS to HRS when further negative bias was applied. When the voltage was varied from 0 to −3 V, the device changes the state from LRS to HRS at ~−1.10 V. The current at −1.10 V was found to be 9 × 10^−7^ A, whereas it was 9 × 10^−5^ A prior to sudden electrical transition from LRS to HRS. The device remained at the HRS state even if the larger negative bias was applied from −1.10 to −3 V or even when the voltage was varied from −3 to 0 V. The device can be triggered again from HRS to LRS state when further 1.10 V was applied at the gate electrode. The set/reset voltage was found to be +/−1.10 V, which is much lower when compared with other ferroelectric or oxides based resistive memory devices where set/reset voltage of ~5 V was required^6^,^8^,^9^,^10^. The obtained low set voltage in this study may lead to low-power dissipation. It is noteworthy that the set and reset processes in our devices are free from the formation of conductive bridge filaments or any other electroforming process. Since, large current and voltage are required for inducing such a forming process in other oxides or solid electrolytes based resistive NVM devices, our present approach saves the devices from thermal damage and thereby enhances the reliability of the devices^11^.

Several carrier injection mechanisms are responsible for these resistive switching memory devices: (i) Ohmic conduction where I ∞ V, (ii). Thermionic emission where lnI ∞ V, (iii) Space charge limited current where I ∞ V^α^ where α ≥2, (iv) Poole-Frenkel emission where ln(I/V) ∞ V^1/2^, and (v) Fowler-Nordheim tunnelling, where I ∞ V^2^ exp (-E_a_/V), where E_a_ is the kinetic energy of the charge carriers^41^. In our devices, the conduction mechanisms were determined by fitting both the HRS and LRS I-V curves and are shown in Supplementary Fig. S1 online. During HRS, the Ohmic conduction was responsible for current transport when the voltage was varied from 0 to 0.5 V. However, space charge limited conduction (SCLC) mechanism comes into play when the higher voltage was applied from 0.6 to 3 V. During LRS, both the thermionic (TE) and thermionic field emission (TFE) were responsible at low voltages (from 0 to 0.5 V). At higher voltages (1 to 3 V), SCLC conduction mechanism dominates the current transport in the devices. To investigate the device switching behaviours, the devices were measured in pulse mode to demonstrate the data storage capability (as shown in Fig. 5(b)). Initially the device was in the virgin state (or HRS state). After applying a positive pulse (3 V amplitude for 0.3 s), the device was set to LRS from HRS. The device was again set back to HRS from LRS when a negative pulse (−3 V amplitude for 0.3 s) was applied across the devices. The HRS is termed as off state, whereas, the LRS is referred as on state. Both the on and off states were read by applying 0.7 V bias continuously for 10 s. The switching characteristics of our fabricated devices were observed for 100 s by applying positive and negative pulses. The obtained results indicate the effectiveness of our devices for resistive switching purpose.

To further understand the efficacy of BT-BFO for resistive switching, the devices were measured where BT-BFO was absent between the Pt electrode and the Nb:STO. The current across the Pt/Nb:STO device is found to be linear and no hysteresis or switching behaviour was observed even with the applied voltages from 0 to 3 V, 3 to 0 V, 0 to −3 V, and −3 to 0 V (as shown in Fig. 5(c)). Similar linear I-V characteristics of Pt/Nb:STO was also obtained by Zhang *et al.*^42^ Therefore, the origin of switching obtained in our fabricated devices is due to the presence of BT-BFO between the Pt and the Nb:STO substrate. The ON/OFF ratio is explained as the ratio between the ON current and the OFF current. For high performance NVM devices, high ON/OFF ratio is always desirable for achieving high reliability and throughput. Figure 5(d) shows the ON/OFF ratio in Pt/BT-BFO/Nb:STO memory devices at different voltages from 0 to 3 V, where the transition voltage is also present when the current state was suddenly changed from HRS to LRS. The variation of ON/OFF ratio at different voltages might be useful for multilevel storage purposes^16^,^17^. However, it is interesting to note that the maximum ON/OFF ratio was found to be 1000 at 1.10 V, which is also almost similar to that of the coercive voltage. Moreover, if we superimpose the I-V characteristics with the hysteresis characteristics obtained from PFM and the electrical P-V characteristics, the switching voltage coincides in either direction. Therefore, one can conclude that the device switching properties are related to the polarization direction of the BT-BFO ferroelectric material^43^. The obtained ON/OFF ratio from our fabricated devices is believed to be high when compared with other ferroelectric NVM devices^8^,^9^,^44^. Even nanostructures of BFO provides less ON/OFF ratio (~500)^7^. Although the ON/OFF ratio was found to be ~10^4^ for Pt/BFO/Nb:STO memristors as reported by Hu *et al.*^10^, the authors did not measure the ON/OFF ratio over a wide voltage range, which is required for multi-bit operation. It is noteworthy to mention that our devices exhibited lower set voltage compared with Hu *et al.*^10^, thus they present a stronger potential for low-power applications.

**Figure 6:**
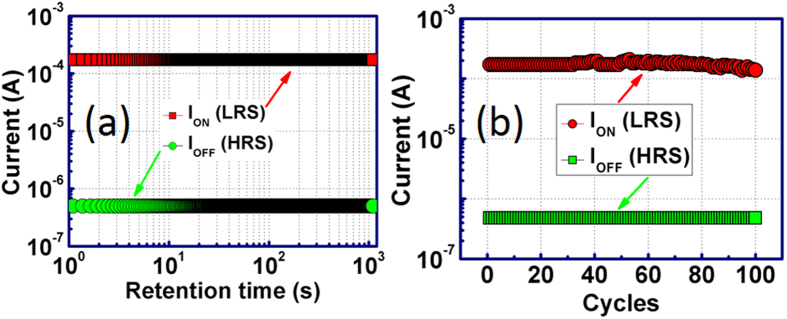
Charge retention properties. (**a**) Retention and (**b**) endurance characteristics in BT-BFO based resistive NVM devices. Stable retention characteristics are observed for 1000 s. The cyclic endurance measurements show the reliability of the devices.

To further elucidate the potential application of Pt/BT-BFO/Nb:STO NVM devices, we have performed the measurement of retention and endurance characteristics. The retention characteristics were measured by biasing the devices at 1 V and the corresponding result is shown in Fig. 6(a). From this figure, it can be seen that the device exhibits stable charge retention time for 10^3^ s. No degradation in the on or off current was observed and the device maintains almost same ON/OFF ratio of 1000 throughout the experiments. To further demonstrate the reliability of these devices, we have performed endurance measurements for 100 cycles by biasing the devices at 1 V. The device maintains high ON/OFF ratio up to 80 cycles with no degradation (as shown in Fig. 6(b)). Therefore, the Pt/BT-BFO/Nb:STO structures show stable switching characteristics and demonstrate the potential for NVM application.

### Band alignments study

**Figure 7:**
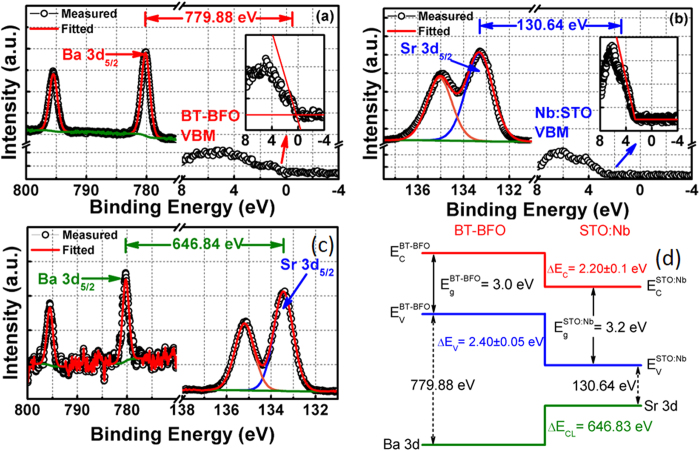
Determination of BT-BFO/Nb:STO heterojunction band alignments using x-ray photoelectron spectroscopy (XPS). XPS spectra of (**a**) Ba 3d CL and VBM of 50 nm BT-BFO thin films on Nb:STO; (**b**) Sr 3d CL spectrum and VBM of Nb:STO; (**c**) Ba 3d and Sr 3d CL spectra of 1.5 nm thin BT-BFO/Nb:STO interface; and (**d**) Energy banddiagram of BT-BFO/Nb:STO heterointerface obtained from XPS measurements. The banddiagram between BT-BFO and Nb:STO offers the Staggered type heterostructure.

To gain insight into the device operation principle and electron transport properties across the BT-BFO/Nb:STO heterointerface, we conducted the band alignment measurement between the BT-BFO and the Nb:STO using XPS technique. The bandgap of BT-BFO was found to be 3 eV [shown in Supplementary Fig. S2 online]. The material compositions of BT-BFO and Nb:STO were confirmed with the XPS (shown in Supplementary Fig. S3 online) and SIMS techniques (shown in Supplementary Fig. S4 online). Three different samples were prepared to determine the band alignment properties of BT-BFO/Nb:STO heterojunction: (1) 40 nm (thick) BT-BFO on Nb:STO substrate, (2) Nb:STO substrate, and (3) 1.5 nm (thin) BT-BFO on Nb:STO substrate. The Ba 3d core-level (CL) spectrum and valence band maximum (VBM) of BT-BFO film are shown in Fig. 7(a), which was obtained from sample (1). The energy difference between the Ba 3d centroid and the BT-BFO VBM was found to be 777.19 ± 0.05 eV. Figure 7(b) shows the Sr 3d CL spectrum and the VBM of Nb:STO film, which was obtained from sample (2). The energy difference between the Sr centroid and the Sr VBM was found to be 14.36 ± 0.05 eV (as shown in Fig. 7(b)). Figure 7(c) shows the Ba 3d CL and the Sr 3d CL spectrum of 1.5 nm BT-BFO on Nb:STO, obtained from sample (3). For this BT-BFO/Nb:STO heterointerface, the energy difference between the Ba 3d centroid and the Sr CL was found to be 762.86 ± 0.05 eV. The valence band offset (*ΔE*_*V*_) at the BT-BFO/Nb:STO heterointerface was determined from the following equation (1)^45^,^46^


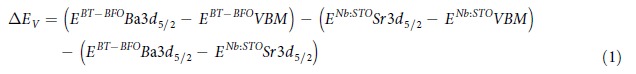


The conduction band offset (*ΔE*_*C*_) at the BT-BFO/Nb:STO interface was calculated from the following equation (2),


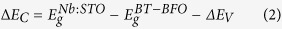


where 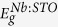
is the bandgap of Nb:STO and the value of 3.20 eV was obtained from the literature^31^ and 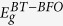
is the bandgap of BT-BFO which was determined to be 3 eV (Supplementary Fig. S2 online). The *ΔE*_*V*_ for BT-BFO/Nb:STO heterointerface was found to be 2.40 ± 0.05 eV, whereas the *ΔE*_*C*_ of BT-BCN/HfO_2_ interface was calculated to be 2.20 ± 0.1 eV. Figure 7(d) shows the schematic of the band alignment of BT-BFO and Nb:STO heterojunction based on the obtained band alignment results and it was found to be type-II staggered gap.

**Figure 8:**
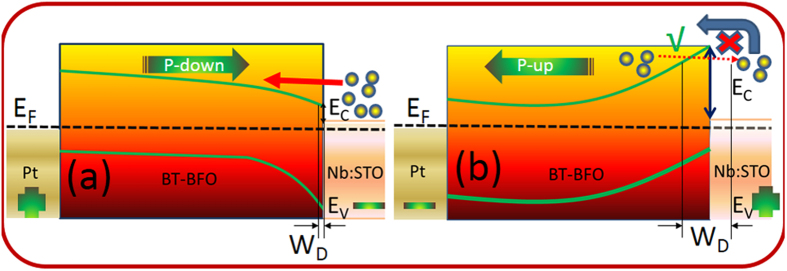
Schematic energy banddiagrams of a Pt/BT-BFO/Nb:STO NVM devices. (**a**) When positive voltage was applied at the Pt, top electrodes, drive the device to LRS; and (**b**) When negative voltage was applied at the Pt top electrodes, drive back the device to HRS.

The resistive memory effect can be further understood by examining the energy band-diagram of Pt/BT-BFO/Nb:STO devices with the presence of positive and negative bias. Based on the band alignment shown in Fig. 7(d), we can consider Nb:STO as a n-type and the BT-BFO layer as a p-type semiconductor. Therefore, a depletion region and a potential barrier form across this p-n junction when BT-BFO and Nb:STO layers are in contact^5^,^7^,^10^. When a positive voltage (3 V) was applied at the Pt electrode (as shown in Fig. 8(a)), the ferroelectric polarization in the BT-BFO is downward. This positive polarization in the BT-BFO induces polarization field, which attracts majority carrier electrons from the Nb:STO and repels majority carrier holes from the BT-BFO, creating decrease in the depletion region at the BT-BFO/Nb:STO heterointerface. Moreover, the polarization field also attracts oxygen ions from the Nb:STO and the band bends downward, resulting in a decrease in the barrier height. Thus, the device sets to a stable LRS state. On the other hand, when a negative voltage (−3 V) was applied at the Pt electrode (as shown in Fig. 8(b)), the ferroelectric polarization in BT-BFO is upward. This negative polarization in BT-BFO also induces polarization field which repels majority carrier electrons from the interface toward Nb:STO and attracts majority carrier holes toward BT-BFO, causing increase in the depletion region at the BT-BFO/Nb:STO interface. As the band bends upward, oxygen ions will be restricted to move from Nb:STO to BT-BFO. Thus, the device switches from a LRS to HRS^5^,^7^,^10^. Therefore, from the band-diagrams, it was found that the ferroelectric polarization and the resistive states are related to the modulation of depletion barriers in Pt/BT-BFO/Nb:STO devices.

### Study of photovoltaic properties

**Figure 9:**
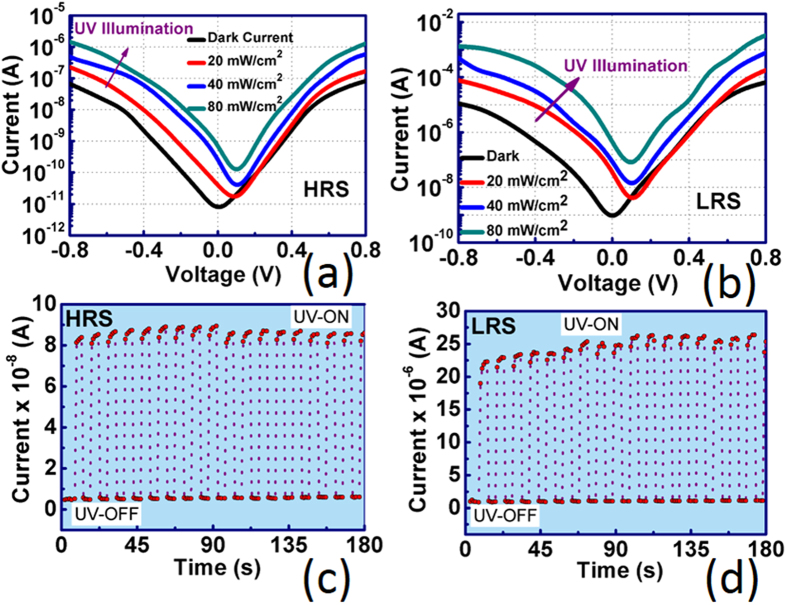
I-V characteristics of the BT-BFO based UV-detector under different light intensity. (**a**) Polarization up and (**b**) down states. Transient measurement of the ON/OFF current of the device for (**c**) polarization up and (**d**) down states.

To establish the photovoltaic characteristics of BT-BFO, we have excited the electrons in the BT-BFO/Nb:STO heterojunction system with UV-light which has an energy greater than the bandgaps of materials used here (3 eV for BT-BFO and 3.2 eV for Nb:STO). The UV-photovoltaic effect was determined using a UV-light wavelength of 253.7 nm at different intensities. When the device is irradiated with the UV light, the electrons from the valence band reaches to the conduction band in BT-BFO material. The electrons then cross the potential barrier at the BT-BFO/Nb:STO interface and reach the Nb:STO material. At the same time, the holes are arriving at the BT-BFO from the Nb:STO material, thus providing photocurrent. Figure 9(a,b) show the I-V characteristics in the dark and with UV light irradiation for the HRS and LRS state, respectively. The current increased significantly from the dark current when the device was exposed with UV light. During the HRS and LRS state, the current follows the same trend with UV light, only the difference is in their magnitude. The photocurrent depends on the depletion layer width of the heterojunction^31^ and the depletion layer width for LRS is less than HRS. Thus, the photocurrent at LRS was found to be larger than the photocurrent obtained in HRS. It was also observed that in both LRS and HRS, the photocurrent depends on the intensity of the light. Increasing light intensity can generate more electron-hole pairs; therefore, the current increases with the increase in light intensity. For HRS, the dark current was found to be 2.20 × 10^−9^ A at 0.4 V and increases from 1 × 10^−8^ to 1 × 10^−7^ A at same voltage when the device was irradiated with 80 mW/cm^2^ UV light. Whereas for LRS, the dark current was 6.56 × 10^−7^ A at 0.4 V and increases from to 2.59 × 10^−6^ at same voltage when the device was irradiated with 80 mW/cm^2^ UV light. Figure 9(c,d) show the time dependent photocurrent with the UV light ON and OFF for the LRS and HRS, respectively. The ON/OFF states are clearly distinguishable from this transient measurement. The rise and fall times can be deduced from these two expressions 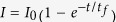 and 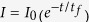, respectively^47^. For LRS, the rise and fall times were found to be 0.65 s and 0.72 s, respectively. Whereas for HRS, the rise time and fall times were found to be 0.77 s and 0.82 s, respectively. The fast rise and fall time indicates the efficient UV-photo detection from BT-BFO/Nb:STO staggered gap heterojunction structure.

### High-low frequency rectifying properties

**Figure 10:**
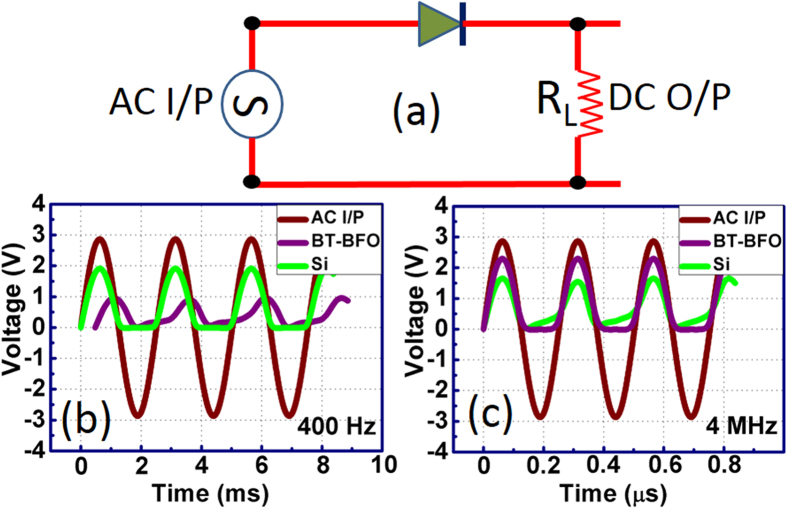
Low-high frequency rectifying properties. (**a**) Circuit diagram of a BT-BFO based rectifier device. Input signal amplitude was maintained constant at 3 V. The output characteristics of the fabricated devices were recorded operated at a frequency of (**b**) 400 Hz and (**c**) 4 MHz input ac signals and compared with a commercial Si rectifier diode (1N4005).

Figure 10 shows the rectification performance and frequency response from the fabricated Pt/BT-BFO/Nb:STO devices. The performance was compared with the commercial Si p-n junction diode (1N4005) in order to evaluate the capability of BT-BFO for rectification and high-frequency signal processing. The half-wave rectifier was constructed using the simple circuit shown in Fig. 10(a), where ac signal (frequency 400 Hz, 4 MHz and 3 V peak-to-peak amplitude) was applied at the input and the output signals were recorded across a 100 Ω load-resistance. Both the BT-BFO and Si device show half-wave rectified signal at the output. At 400 Hz, the rectified signal amplitude is higher for Si 1N4005 device compared with our fabricated BT-BFO device (as shown in Fig. 10(b)). In addition, there is a phase lag in BT-BFO device with respect to the input signal. However, at high frequency (4 MHz), the phase lag disappeared for the BT-BFO device and the rectified signal amplitude is also improved (as shown in Fig. 10(c)). Our results are highly promising and these can be compared to other reports based on organic-inorganic hybrid diode and carbon-nanotube diode based rectifier for high-frequency signal processing^48^,^49^. Thus, our fabricated device can be employed as an electrical switch to direct the pixels in low or high frequency images. In order to test the reliability of BT-BFO based memory devices, the devices were kept under low vacuum (~10^−3^ torr) for one month in dark environment at room temperature and then retested. The rectified signal remains almost identical, thus, the aging time has no bearing effect on the long term storage stability of our fabricated Pt/BT-BFO/Nb:STO based NVM devices.

## Summary

In summary, we have demonstrated the resistive non-volatile memory effect of lead-free BT-BFO ferroelectric epitaxial thin-films grown on semiconducting Nb:STO substrates. The device operates at low voltage of ±1.10 V and offers high ON/OFF ratio of 1000, fast switching and excellent retention and endurance properties. The band alignment between the BT-BFO and the Nb:STO heterojunction was found to be type-II staggered type, and the operating principle and electrical transport properties of memory devices were explained based on the *ΔE*_*C*_ and *ΔE*_V_ values. The BT-BFO/Nb:STO memory devices suppresses the cross-talk between adjacent memory cells due to the use of semiconducting Nb:STO substrate instead of metal electrode^41^. Moreover, the BT-BFO/Nb:STO heterojunction is found to be an efficient detector for UV-detection exhibiting fast response. The half-wave rectifier circuit was constructed using BT-BFO and it shows an excellent performance in presence of low and high-frequency signals. The obtained results provide a new pathway towards design of future generation multifunctional electronic devices such as low-power and fast switching ReRAMs, optoelectronic detectors, and high-frequency signal processors. Furthermore, the BT-BFO material shows excellent photovoltaic properties; therefore, the optical read-write ultrafast memories can also be realized.

## Methods

### Materials synthesis and device fabrication

High-quality BT-BFO epitaxial films were grown on one-sided polished (100)-oriented Nb:STO single crystal substrates by PLD from a high purity BT-BFO target. The target of (1-x)BaTiO_3_ – xBiFeO_3_ (x = 0.725) was synthesized using the conventional solid state reaction method. For this, stoichiometric amounts of TiO_2_, Bi_2_O_3_, Fe_2_O_3_, BaCO_3_ (from Ward Hill, USA) were ball milled under ethanol for 24 h followed by drying at 80 °C for 6 h. The obtained powder was calcined at 1000 °C for 2 h followed by ball milling for 48 h under ethanol. Then the powder was pressed into a cylindrical target using a uniaxial press followed by isostatic pressing to achieve high green density. The cylindrical target was sintered at 1350 °C for 2 h to achieve highly dense (>95.5%) body. Nb-(0.7 wt%) doped SrTiO_3_ was used as a substrate and it was degreased by acetone and 2-propanol, and finally rinsed in deionized water for 1 min. The BT-BFO (100) thin-films were deposited by PLD technique using a KrF excimer laser (λ = 248 nm) on Nb:STO at a deposition rate of 0.5 Å/s using the synthesized BT-BFO target. The focused laser beam irradiates the rotating target at 89 rpm with a laser energy density of ∼2.5 J/cm^2^ at a repetition rate of 10 Hz in an oxygen pressure of 300 mTorr and the temperature was maintained at 800 °C during the deposition process. The thickness of BT-BFO film was found to be 50 nm. Pt top electrodes (area 600 μm × 600 μm) and In bottom electrodes were deposited using Kart J. Lesker PVD 250 electron beam evaporator. The purpose of In deposition at the bottom of Nb:STO was to obtain low contact resistance. For UV-photodetectors, In was served as a top and bottom electrodes.

### Characterizations

To investigate the crystalline structure of deposited BT-BFO, XRD pattern was recorded using a PANalytical X’Pert Xray diffractometer (Cu Ka radiation) with PIXel3D detector at an operating voltage of 45 kV and a current of 40 mA. The bandgap of BT-BFO film was determined from transmission spectra obtained by HitachiU-4100 UV-vis-NIR spectrophotometer. All the electrical measurements were conducted at room temperature using Keithley 4200 semiconductor characterization system. The band alignments between the BT-BFO and the Nb:STO were investigated using a PHI Quantera SXM XPS system with a monochromated Al Kα (energy of 1486.7 eV) X-ray source. All binding energy spectra were collected with a pass energy of 26 eV and an exit angle of 45°, including the CL spectra as well as the angle-integrated photoelectron energy distribution curves for the VBM. All binding energy spectra collected were adjusted to the adventitious carbon peak – C1s – CL at 285.0 eV. Dynamic secondary ion mass spectrometry (SIMS) was performed using Cameca IMS-7f GEO with Cs+ as primary ion beam to determine the compositional profile of Ba, Ti, O, Bi, Fe, Nb, and Sr atoms in the 300 nm BT-BFO thin films onto Nb:STO substrates. The out-of-plane piezoresponse and local hysteresis loops were measured by PFM using a scanning probe station (Bruker Dimension Icon, USA). The AC drive amplitude was 1000 mV (~300 kHz) during the DC bias sweep. In order to investigate the polarization switching behaviour of domains in BT-BFO, a positive dc bias of 0 and ±6 V was applied on BT-BFO films using a scanning tip (SCM-PIT, Bruker). Ferroelectric P-V measurement was performed using modified Sawyer-Tower Bridge Precision II (Radiant Technologies, Albuquerque, NM). To study the UV photo detection property the current voltage characteristics has been measured by illuminating the sample with an UV lamp (Philips TUV PL-S) having wavelength spectrum of 253.7 nm (UV-C region) and the shorter and longer wavelengths were blocked by a high pass and low pass filter, respectively.

## Additional Information

**How to cite this article**: Kundu, S. *et al.* Lead-free epitaxial ferroelectric material integration on semiconducting (100) Nb-doped SrTiO_3_ for low-power non-volatile memory and efficient ultraviolet ray detection. *Sci. Rep.* **5**, 12415; doi: 10.1038/srep12415 (2015).

## Supplementary Material

Supplementary Information
