# Supplementary material for: Lead-free epitaxial ferroelectric material integration on semiconducting (100) Nb-doped SrTiO3 for low-power non-volatile memory and efficient ultraviolet ray detection
Source: Sci Rep. 2015 Jul 23;5:12415. doi: 10.1038/srep12415 (PMC5378894; doi:10.1038/srep12415)
Supplement: Supplementary Information [file srep12415-s1.pdf]

Supplementary Information

Lead-free epitaxial ferroelectric material integration on semiconducting (100) Nb-doped $SrTiO_3$ for low-power non-volatile memory and efficient ultraviolet ray detection

Souvik Kundu[1,*] Michael Clavel[2], Pranab Biswas[3], Bo Chen[1], Hyun-Cheol Song[1], Prashant Kumar[1], Nripendra N. Halder[4], Mantu K. Hudait[2], Pallab Banerji[3], Mohan Sanghadasa[5], and Shashank Priya[1]

[1]*Center for Energy Harvesting Materials and Systems (CEHMS), Department of Mechanical Engineering, Virginia Tech, Blacksburg, Virginia 24061, USA*

[2]*Advanced Devices & Sustainable Energy Laboratory (ADSEL), Bradley Department of Electrical and Computer Engineering, Virginia Tech, Blacksburg, Virginia 24061, USA*

[3]*Materials Science Centre, Indian Institute of Technology Kharagpur, Kharagpur 721302, India*

[4]*Advanced Technology Development Centre, Indian Institute of Technology Kharagpur, Kharagpur 721302, India*

[5]*U.S. Army Aviation & Missile Research Development & Engineering Center (AMRDEC) Redstone Arsenal, Huntsville, AL 35898, USA*

In our Pt/BT-BFO/Nb:STO devices, the conduction mechanisms were determined by fitting both the HRS and LRS I-V curves and are shown in Fig. S1. During HRS, the Ohmic conduction was responsible for current transport when the voltage was varied from 0 to 0.5 V (Fig. S1(a)). However, space charge limited conduction (SCLC) conduction mechanism comes into play when the higher voltage was applied from 0.6 to 3 V (Fig. S1(b)). During LRS, both the thermionic and thermionic field emission were responsible at low voltages (from 0 to 0.5 V). At higher voltages (1 to 3 V), SCLC conduction mechanism was responsible for the current transport.

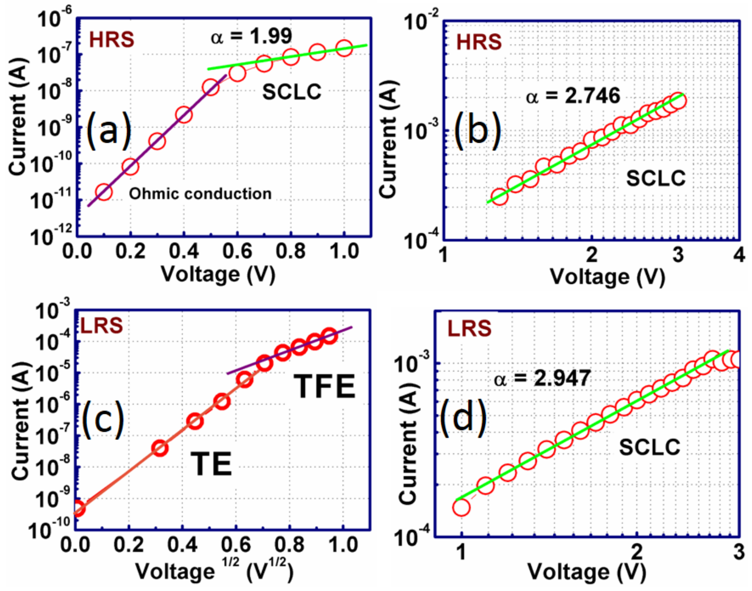

Figure S1 | Transport properties. During HRS, the I-V characteristics of Pt/BT-BFO/Nb:STO (a) from 0 to 0.5 V show that Ohmic conduction was responsible for the current transport. Whereas, from 0.5 to 1 V, the slope 1.99 indicates that the current was due to SCLC. However, from 1 to 3 V, the slope 2.746 depicts that the current is also due to SCLC. On the other hand, during LRS, (c) the I-V characteristics from 0 to 1 V, both the TE and TFE was responsible for the current transport, and (d) from 1 to 3 V, the slope 2.947 represents that the current was mainly due to SCLC.

Figure S2 shows the transmission spectrum of BT-BFO and a sharp absorption peak around 423 nm was found which is attributed to the direct band-to-band transition. Well known Tauc's relationship has been utilized to determine the bandgap of BT-BFO and the value was found to be 3 eV. The XPS survey spectra were recorded from Nb:STO substrates (Fig. S3(a)) and thick BT-BFO (Fig. S3(b)) films for a binding energy range of 0 eV to 800 eV. The Nb-related peaks were absent during XPS scan from Nb:STO, because the doping concentration of Nb (0.7%) is way below the XPS detection limits. To further verify the presence of Nb within the as-deposited BT-BFO, SIMS depth profiling was performed and the presence of Nb was identified (see Fig. S4).

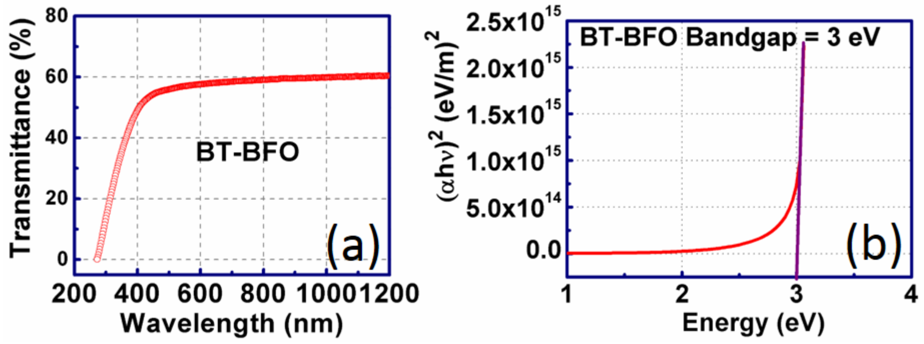

Figure S2 | Optical band-gap studies of BT-BFO thin films. (a) Transmission spectra for BT-BFO thin films deposited on glass substrate. A sharp absorption peak found around 423 nm can be attributed to the direct band-to-band transition; and (b) $(\alpha h\nu)^2$ vs $h\nu$ to determine the optical bandgap of BT-BFO and it was found to be 3 eV.

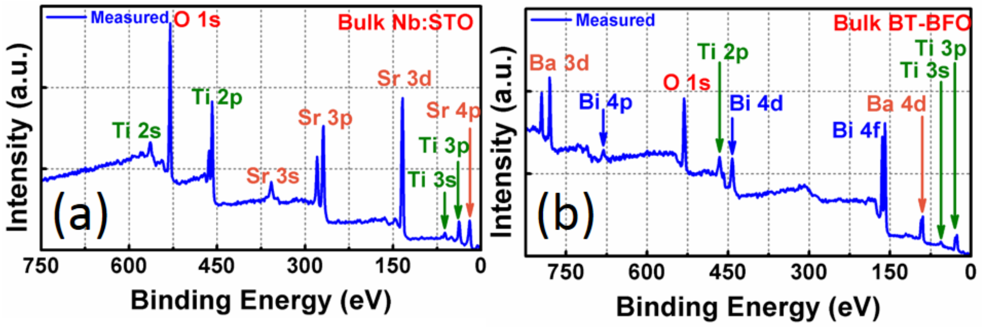

Figure S3 | Surface survey spectra using XPS. (a) Survey spectrum for Nb:STO substrates; and (b) survey spectrum for BT-BFO thin film.

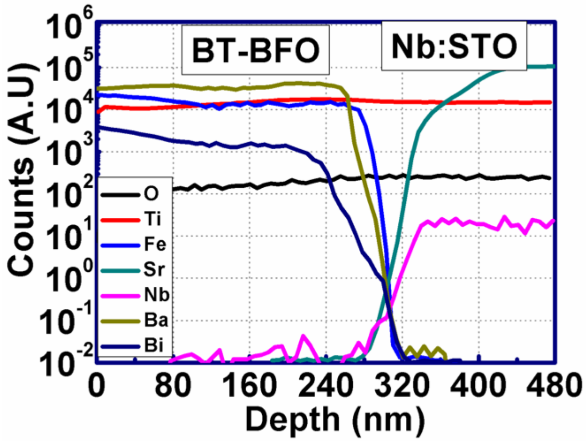

Figure S4 | SIMS depth profile of a 300 nm BT-BFO films onto Nb:STO substrates.
